# In silico and in vivo hepatoprotective activity of the synthesized 5-benzylidene-2-thiohydantoin against diethylnitrosamine-induced liver injury in a rat model

**DOI:** 10.1038/s41598-023-27725-x

**Published:** 2023-03-22

**Authors:** Lana S. Akree, Zahra A. Amin, Hiwa O. Ahmad

**Affiliations:** 1 Department of Pharmacognosy, College of Pharmacy, Hawler Medical University, Erbīl, 44001 Iraq; 2 Department of Pharmaceutical Chemistry, College of Pharmacy, Hawler Medical University, Erbīl, 44001 Iraq

**Keywords:** Chemical biology, Gastroenterology, Molecular medicine

## Abstract

In the present study, the hepatoprotective effect of 5-benzylidine-2-thiohydantoin (5B2T), a unique derivative of the thiohydantoin group, on liver injury induced by diethylnitrosamine (DEN) in male rats was investigated. The experimental animals were divided into three groups, each with 14 rats. Rats in group I were considered to be controls and received only 10% Tween 80. Rats in group II were injected with 200 mg/kg DEN intraperitoneally. Rats in group III were injected with a single dose of DEN 200 mg/kg intraperitoneally and received the treatment orally (50 mg/kg, 5B2T) for two durations, 3 and 6 weeks. At the end of the experiment, blood was collected for the analysis of liver function and pro-inflammatory cytokine IL-6 and tumor necrosis factor α (TNF-α) levels. Additionally, liver specimens were used for histopathological examination and immunohistochemistry. The single intraperitoneal injection of 200 mg/kg DEN into rats resulted in significant elevation of serum enzyme levels of AST, ALT and ALP, which are indicators of hepatocellular damage, along with elevation in TNF-α and IL-6 in the DEN group. The results of both LFTs and ELISA in the treatment group showed improvements and a decline in the levels of the markers. Histopathological examination showed fibrosis, necrosis and infiltration of inflammatory cells in the DEN group, with lower intensity in the treatment group. The results of immunohistochemical staining revealed strong positive staining of both HSA and Ki-67 antibodies in the DEN group, with much lower intensity in the treatment group. The results of the docking study indicated that 5B2T has a remarkable interaction with TNF-α (PDB ID: 1TNF) and human IL-6 (PDB ID: 1IL6) with binding site energies of − 7.1 and − 6.1 (kcal/mol), respectively. The correct absorption and binding between the drug and the receptor was evaluated through computerized molecular docking by using the AutoDock program. The conclusion of the results from the current study reflected the interesting hepatoprotective abilities of 5B2T against DEN-induced hepatocellular damage and cancer in experimental rats.

## Introduction

Liver cancer is one of the most common and critical malignancies worldwide, with rapid growth and poor prognosis. Liver cancer may start as normal liver injury that develops into fibrosis and can then progress to cirrhosis and end in a critical case of carcinoma^1^. Hepatocellular carcinoma (HCC), simply known as liver cancer, is a fatal type of cancer with high morbidity and mortality rates. One reason why the diagnosis of liver cancer is difficult is because of its asymptomatic state until it reaches developed stages^2^. Hepatocellular carcinoma is composed of malignant neoplastic cells that to a large extent look like hepatocytes, which alter with differentiation^3^. The liver is the largest internal organ, and it is responsible for the accomplishment of many vital activities, for instance, the eradication of internal and external biological waste materials and metabolic waste (e.g., bile, urea, and lipids) out of the blood circulation. In addition, it has a very important role in many functions of the immune system^4^. Hepatocellular carcinoma incidences are currently increasing in both developed and developing countries due to the high rates of viral infections (e.g., HBV, HCV and HDV), alcoholism and obesity^5^.

Nitrosamines are a group of toxic chemical compounds that are considered to be very potent, toxic and carcinogenic for both humans and animals^6^. N-nitroso alkyl compounds, specifically diethylnitrosamine (DEN), can initiate different types and stages of malignancies in various organs, including the liver, lungs and blood, and are widely used as inducer chemicals to promote cancers in experimental animals (most commonly rats)^7^. Diethyl nitrosamines were previously well established as preservatives for many foods, such as dairy products, smoked and salted fish and meat, soybeans and alcoholic drinks. In the food industry, some chemicals are added as microbial growth inhibitors, preservative materials, colorants and flavor stabilizers. Most famously, nitrites are used. Nitrites are transformed to nitrosamines under the influence of high temperature and gastric acidic juice, and the net result is that these types of foods are a major source of these toxic materials, which is why these chemicals are no longer utilized as preservatives in food processing^8^. Hydantoins (also known as glycolylurea, arise from the reaction of glycolic acid and urea) and their derivatives (and some other molecules) are a group of heterocyclic compounds (organic), and they are considered to be very important and vital chemicals, because they play a pivotal role in many biological and pharmacological approaches, as well as in medicinal chemistry and in agrochemical applications, in addition to these, they represent the key precursors for the chemical and enzymatic synthesis of many crucial non-natural alpha amino acids and their conjugates of medical importance. Hydantoin is a colorless solid, and is a derivative of the oxidation of imidazolidine, it carries the formula C_3_H_4_N_2_O_2_^9^. Thiohydantoins and their derivatives are a focus of interest for scientists and researchers nowadays because of their high bioactive and therapeutic potentials. Thiohydantoin is a sulfur (S, or thio) analog of the compound hydantoin (also known as imidazolidine-2,4-diones), at which one or two carbonyl groups are replaced by thiocarbonyl groups^10^. One interesting fact about this group of molecules is that their biological activity is changed and affected according to the nature of substituents. Significant numbers of thiohydantoin derivatives can be classically prepared by the condensation reactions of various aldehydes^11,12^. Among the major biological applications, therapeutic activities and pharmacological purposes of this class of heterocyclic compounds include anti-tumor activity, anti-bacterial activity, anti-parasitic activity, anti-malarial activity, anti-fungal activity, anti-epilepsy activity, anti-melanogenesis activity^10^. It's interesting to note the emergence of the thiohydantoin ring as an efficient pharmacophoric ingredient in the development of potent inhibitors for EGFR and VEGFR growth factor receptors. Furthermore, thiohydantoin derivatives are androgen receptor and TNF-antagonists, as well as effective inhibitors of several enzymes, including DNA Topoisomerase I, II (TopI, II), NOXs, isocitrate dehydrogenases (IDHs), B-cell lymphoma-2 (Bcl-2) and sirtuins (SIRTs), kinesin spindle protein (KSP), prolyl hydroxylases 1–3 (PHD 1–3), CDK2, and CDK4.11,25. The aim of the present study was to test the hepatoprotective effect of the new thiohydantoin derivative 5-benzylidine-2-thiohyadantoin (5B2T) in a rat model of DEN-induced liver injury.

## Materials and methods

For the present study, a newly synthesized derivative of thiohydantoin, 5B2T, was prepared experimentally in the laboratory and used as a treatment drug for liver injury.

### Chemistry

All melting points of the compounds were determined on Griffin apparatus. Infrared spectra were recorded in the range 4000–600/cm via a SHIMADZU CORP series FTIR instrument. The NMR spectra were run at Bruker DPX 400 (400 MHz) spectrometer using tetramethylsilane (TMS) as the internal standard. Chemical shifts were measured in ppm (δ) related to TMS (0.00) ppm. High-resolution mass spectrometric data were obtained in electrospray (ES) mode unless otherwise reported, on a Waters Q-TOF micro-mass spectrometer using a Gilson 232XL auto-sampler.

### Preparation of the drug

Commercially available 2-thiohydantoin was placed with the required aldehydes in a round bottom flask equipped with a magnetic stirrer and reflux condenser under a nitrogen atmosphere in triethylamine and water. The mixture was stirred overnight at room temperature, and the pH was adjusted to 3 by adding 3 M HCl. The solid product was filtered off and washed with diethyl ether and water. The pure compounds were collected and dried^13^.

5B2T (the final product) was synthesized by using commercially available hydantoin dissolved in ethanol in the presence of benzaldehyde and triethylamine and in an overnight H2O reflux (this was done following the success of a test reaction).

### Acute toxicity test

The OECD-423 guidelines was followed to determine the safety of the new synthesized compound 5B2T by administration of a single dose of 2 g/kg and 5 g/kg to the experimental mice^14^. In brief, 36 healthy mice (18 males and 18 females) were divided into three groups labeled as: vehicle (dH_2_O), low dose (2 g/kg) and high dose (5 g/kg) of 5B2T, respectively. Each mice was made to fast an overnight prior to dosing. Food was withheld for another 3–4 h after the administration. The animals were closely observed for 30 min and at 2, 4, 24 and 48 h after the administration for detection of any signs of acute clinical toxicity, morbidity and mortality. Behavioral observations include: respiration (dyspenea), salivation, skin piloerection, exopthalmus, convulsion and locomotion changes. After keeping alive for 14 days, on day 15, the mice were sacrificed to measure serum biochemical (liver and kidney) parameters following the standard methods^15^.

### In vivo animal model

Forty-two healthy adult male rats weighing between 200 and 250 g with an average age between 3 and 4 months were used in this study. They were obtained from the animal house unit/College of Pharmacy/Hawler Medical University. The animals were given standard food and tap water. The animals were handled and received human care according to the ethical principles of the National Institutes of Health's Guide for the Care and Use of Laboratory Animals^16^ under the permission of the Ethics committee of College of Pharmacy/Hawler Medical University (no. 2021.25.08-205 HMU. PH. EC). All methods are reported in accordance with ARRIVE guidelines (https://arriveguidelines.org). The animals were maintained at 22 ± 3 °C under a 12 h–12 h light/dark cycle with 50–60% humidity for at least one week prior to the experiment. Rats were grouped into three groups (n = 12). The administration of the dosage was done considering the body weight (B.W.) of the rats. Group I was given 10% Tween 80 throughout the experiment and was regarded as the placebo group. Rats in group II received a 200 mg/kg^6^ single-dose intraperitoneal injection of DEN following the method of Rezaie et al.^6^ and served as liver injury positive control group, while rats in group III were given 50 mg/kg 5B2T as an oral treatment in two periods: the first period lasted for 3 weeks, and the second period lasted for 6 weeks. At the end of the experiment, all rats were sacrificed, and blood was collected for biochemical analysis and proinflammatory cytokine analysis by ELISA. The tissues of the harvested livers of the rats were fixed with 10% formalin and then subjected to a series of hydration and dehydration reactions. Then, they were embedded, sectioned, processed and stained with hematoxylin and eosin stains and tested for detection of specific antibodies, which were nuclear protein (Ki-67) and hepatocyte specific antigen (HSA).

### Molecular docking

**Figure 1:**
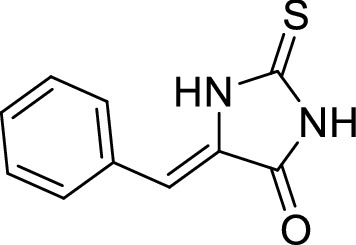
5-benzylidine-2-thiohydantoin.

The two-dimensional (2-D) structures of the ligand molecules (Fig. 1) were built using chemdraw professional 16.0 and converted to 3-dimensional (3-D) structures using Chem3D 16.0 module and saved as a pdb format structures (http://www.cambridgesoft.com/). The ligand was optimized by adding Geister charges and hydrogen and the pdbqt format of the ligands were prepared with AutoDock Tools 1.5.7.

The ligand molecules were then used as input for AutoDock Vina (https://vina.scripps.edu/) to carry out the docking simulation.

The X-ray crystal structure of the target of necrosis factor TNF-α (PDB ID:1TNF) and Human interleukin-6 IL-6 (PDB ID: 1IL6) were retrieved from the RCSB Protein Data Bank web server (http://www.rcsb.org/pdb/). The active binding sites were identified using Discovery Studio visualizer 2021. The grid dimensions were set at 8.41 × 64.05 × 31.20 (PDB ID:1TNF), and 3.49  ×  − 3.45 × 0.44 (PDB ID: 1IL6) according to the coordinates x, y, and z, for the target active binding sites identified in Discovery Studio visualizer 2021. The water molecules were removed from the receptors and polar hydrogen and Kollman charges were added. The pdbqt format of the receptors were generated by AutoDock Tools 1.5.7. AutoDock Vina was compiled and runs under Windows 10.0 Professional operating system. Discovery Studio 2021 was used to deduce the pictorial representation of the interaction between the ligands and the target protein.

The binding affinity (ki) of ligands for selected targets were calculated were assessed using Eq. (1)

1 $$Ki = {\varvec{e}}^{{\left( {\Delta G /\left( {Rx t} \right)} \right)}}$$

where $\Delta G$ is the binding energy in kcal/mol, the universal gas constant R = 1.987 kcal/K/mol, at room temperature (25 °C) T = 273 + 25 = 298 K. Ki is the inhibition constant where the Ki principally depends on the binding (or association) constant (Kb) having a unit of mM^17^.

### ADME prediction

Prediction of pharmacokinetics and physicochemical parameters plays a key role in drug design^18^. The evaluation of drug-likeness properties were evaluated for 5BT using the SwissADME (http://www.swissadme.ch/) and admetSAR (http://lmmd.ecust.edu.cn/admetsar2).^19^ Drug-like molecule must obey the Lipinski’s rule of five (RO5): the molecular weight MW of the active oral drug should be ≤ 500 Da; the log p should be < 5; the number of hydrogen bond acceptors should be nOH ≤ 10; the number of hydrogen bond donors nOHNH should be ≤ 5; and the number of rotatable bonds should be ≤ 10^20^.

## Results

### Spectral analysis of 5B2T

Utilizing the method of preparation mentioned in the materials and methods section, thiohydantoin (2.0 g, 17.2 mmol), trimethylamine (4.9 ml, 37 mmol) and benzaldehyde (1.9 ml, 19 mmol) were added to 50 ml of water. Yield = 69.9%, Mp 270–272 °C, HRMS calculated for C10H8N2OS m/z [M + H] + 204.0358; found 204.0358; 1H-NMR (400 MHz, DMSO-d6): δ 12.41 (s, H, NH), δ 12.19 (s, H, NH), δ7.74 (d, J = 7.4 Hz, 2H, phen), δ 7.45–7.37 (m, 3H, phenyl), δ 6.49 (s, H, CH=C). 13C-NMR (101 MHz, d6-DMSO): δ 177.3 (C=S), δ 164.0 (CO), δ 130.5 (C=CH), δ 128.6 (2xCH), δ 127.3 (C), δ 127.0 (2xCH), δ 126.0 (CH), δ 109.7 (CH=C). IR (neat): vmax = 3225/cm (NH), 1723 cm-1 (C=O), 1475/cm (C=S), 1643/cm (C=C).

### Acute toxicity test

The experimental mice treated with high doses (2 g/kg and 5 g/kg) of 5B2T remain living for 14 days with active healthy condition and there were no obvious toxicity signs and no cases of death were recorded. The results obtained from blood biochemical tests did not demonstrate any difference between the treated groups and the control group as shown in Tables 1 and 2 suggesting that 5B2T was safe when administered orally and the lethal dose (LD_50_) for both genders was greater than 5 g/kg.

**Table 1:** Renal function test of experimental mice treated with high doses of 5B2T.

Groups	Blood urea (mg/dL)	BUN (mg/dL)	Creatinine (mg/dL)	Uric acid (mg/dL)	Serum protein (mg/dL)	Serum albumin (mg/dL)
Male mice						
Vehicle	20.1 ± 0.8	24.7 ± 0.01	1.2 ± 1.8	6.5 ± 0.4	6.0 ± 2.3	3.5 ± 0.3
2 g/kg 5B2T	21.5 ± 1.6	24.3 ± 0.07	1.3 ± 1.1	6.4 ± 0.2	6.1 ± 2.1	3.6 ± 0.2
5 g/kg 5B2T	20.2 ± 0.8	23.8 ± 0.08	1.2 ± 1.6	6.3 ± 0.5	6.5 ± 0.9	3.5 ± 1.2
Female mice						
Vehicle	20.0 ± 0.1	24.1 ± 0.1	1.1 ± 0.6	6.2 ± 1.3	6.3 ± 0.1	3.4 ± 2.7
2 g/kg 5B2T	21.0 ± 0.3	24.8 ± 2.1	1.1 ± 0.7	6.4 ± 0.1	6.1 ± 0.1	3.5 ± 1.3
5 /kg 5B2T	22.2 ± 0.7	24.2 ± 2.2	1.1 ± 0.3	6.5 ± 0.5	6.1 ± 0.1	3.3 ± 0.9

Values expressed as mean ± S.E.M. There are no significant differences between groups. Significant value at p < 0.05.

**Table 2:** Liver function test of experimental mice treated with high doses of 5B2T.

Groups	Total Billirubin (mg/dL)	Direct Billirubin (mg/dL)	Indirect Billirubin (mg/dL)	AST (U/L)	ALT (U/L)	ALP (U/L)
Male mice						
Vehicle	1.1 ± 0.8	0.1 ± 0.2	0.2 ± 0.3	25.4 ± 0.1	33.1 ± 0.3	100.5 ± 0.2
2 g/kg 5B2T	1.0 ± 0.1	0.1 ± 0.2	0.3 ± 0.1	24.9 ± 0.1	34.1 ± 0.6	110.6 ± 0.1
5 g/kg 5B2T	1.0 ± 0.2	0.2 ± 0.1	0.2 ± 0.2	25.0 ± 0.3	33.5 ± 0.9	109.3 ± 0.4
Female mice						
Vehicle	1.2 ± 0.2	0.1 ± 0.3	0.2 ± 0.1	22.2 ± 1.1	36.3 ± 0.3	110.4 + 0.7
2 g/kg 5B2T	1.2 ± 0.3	0.1 ± 0.1	0.2 ± 0.3	23.5 ± 0.8	36.0 ± 0.0	111.5 ± 0.3
5 /kg 5B2T	1.2 ± 0.1	0.2 ± 0.3	0.2 ± 0.3	22.5 ± 0.6	36.1 ± 0.1	111.3 ± 0.3

Values expressed as mean ± S.E.M. There are no significant differences between groups. Significant value at p < 0.05.

### The in vivo hepatoprotective effect

**Figure 2:**
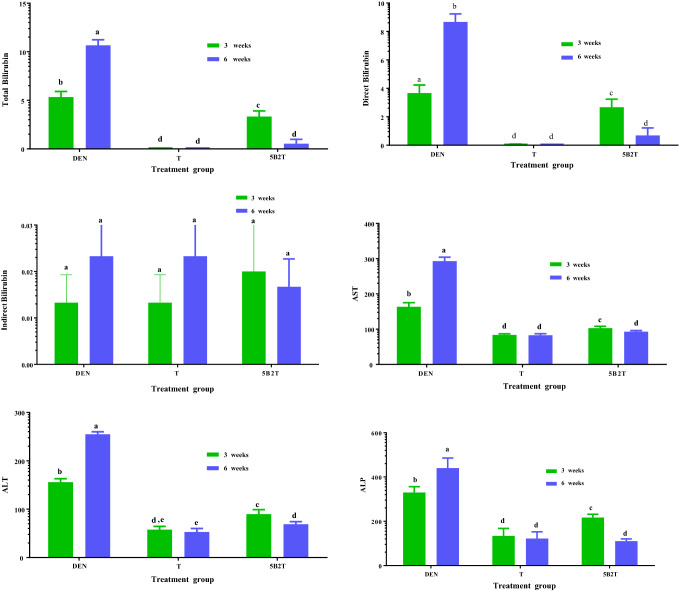
The effect of 5B2T on Liver biochemical parameters. The X axis present the treatment groups and the Y axis presnt the liver function parameters, DEN: diethylnitrosamine, T: 10%tween 80, 5B2T: treatment group. AST: acetate aminotransferase, ALT: alanine aminotransferase, ALP: alkaline phosphatase.

**Figure 3:**
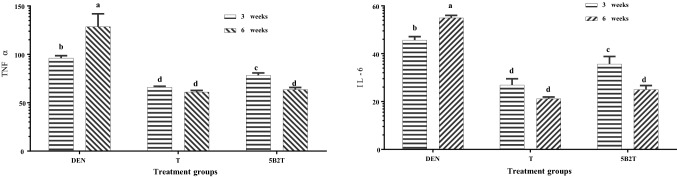
The effect of 5B2T on the Pro-inflammatory cytokines TNF-α and IL-6 levels. The X axis present the treatment groups and the Y axis presnt the cytokines levels, DEN: diethylnitrosamine, T: 10%tween 80, 5B2T: treatment group. TNFα: tumor necrosis factor alpha and IL-6: Interleukin 6.

The results of the present study (Fig. 2) showed that the administration of DEN caused a significant elevation in the biochemical parameters in the first group (DEN-treated rats), while treatment with 5B2T caused a notable decrease in the same biochemical markers. At the same time, the ELISA results reflected a significant increase in proinflammatory cytokines (TNF-α and IL-6) in rats treated with DEN and particularly lower levels of the same cytokines in 5B2T-treated rats (Fig. 3).

**Figure 4:**
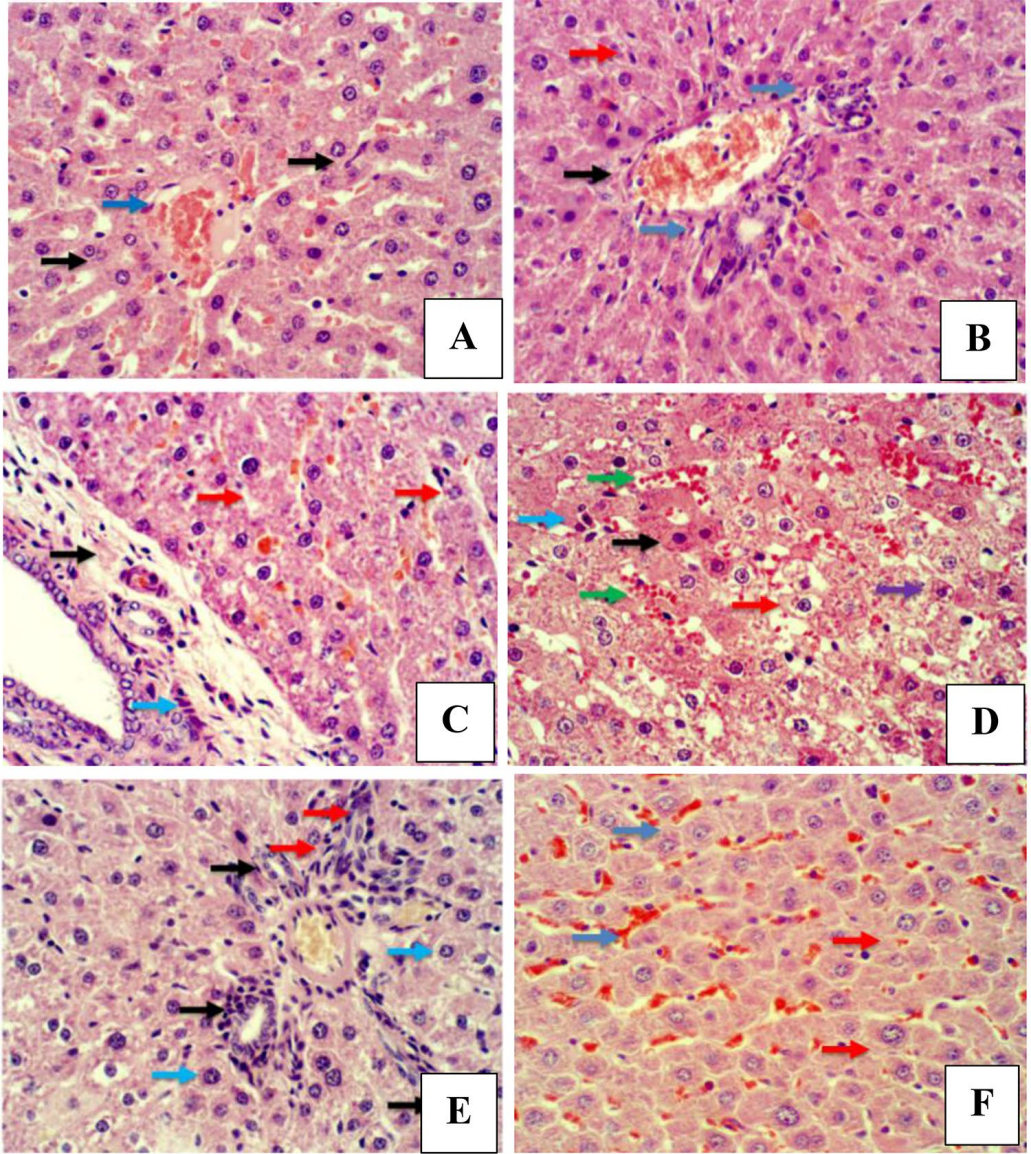
Liver section, H&E. 400×. (**A**) Placebo group/3 weeks, shows normal histological features of hepatic cords (black arrow), and central vein (blue arrow). (**B**) Placebo group/ 6 weeks, shows normal histological features of blood vessels in portal area (black arrow), and bile cuniculi (blue arrow), hepatocytes (red arrow). (**C**) DEN group/3 weeks, shows fibrosis around portal area (black arrow), hyperplasia of Kupffer cells (blue arrow), and dilatation of sinusoids (red arrow). (**D**) DEN group/6 weeks, shows hepatocellular adenoma (black arrow), hyperplasia of Kupffer cells (blue arrow), vacuolar degeneration (red arrow), coagulative necrosis (purple arrow), with dilatation of sinusoids (green arrow). (**E**) 5B2T group/3 weeks, shows hyperplasia of bile cuniculi (black arrow), necrotic hepatocytes (blue arrow), and infiltration of inflammatory cells (red arrow). (**F**) 5B2T group/6 weeks, ShowS mild peliosis (blue arrow), and few hepatocytes showed cellular degeneration (red arrow).

The histopathological examination of the liver sections in the placebo (10% Tween 80)-treated rats showed completely normal histological features for both durations (3 weeks and 6 weeks) (Fig. 4A and B). The administration of DEN for 3 weeks showed highly noticeable liver damage, which was represented by massive disfiguration in hepatic structure, dilation in the sinusoids and massive fibrosis around the portal area (Fig. 4C). The administration of DEN for 6 weeks in turn caused massive abnormal histological features of the hepatic sections, accompanied by infiltration of inflammatory cells around the central vein area, which is an indicator of inflammation and advanced stage of liver injury. Furthermore, dilation of bile canaliculi was presented, along with hepatic peliosis and adenoma (Fig. 4D). Rats treated with DEN and 5B2T showed dramatic improvements in the histopathological appearance of the tested liver sections for both durations (3 and 6 weeks) (Fig. 4E and F).

**Figure 5:**
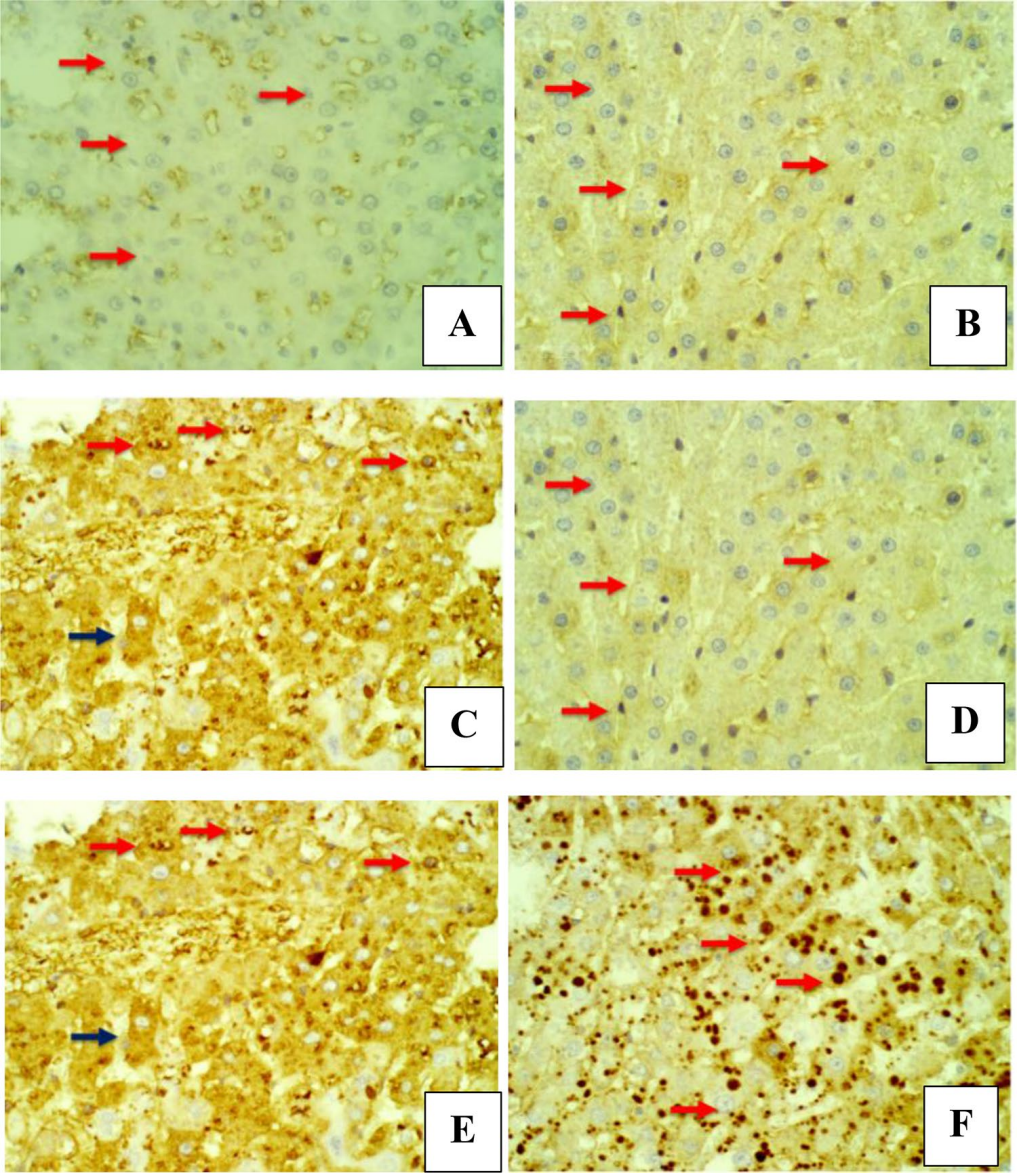
Liver section, IHC *Ki67* -ab. 400×. (**A**) Placebo group/3 weeks, shows negative staining with *Ki67* antibodies in the nucleus of hepatocytes (red arrow). (**B**) Placebo group/6 weeks, shows negative staining with *Ki67* antibodies in the nucleus of hepatocytes (red arrow). (**C**) DEN group/ 3 weeks, shows positive staining with *Ki67* antibodies in the affected hepatocytes which stained the nucleus as dark brawn color (red arrow), notice unspecific staining in other cellular elements (black arrow). (**D**) DEN group/6 weeks, shows positive staining with *Ki67* antibodies in the affected hepatocytes which stained the nucleus as dark brawn color (red arrow). (**E**) 5B2T group/3 weeks, shows few positively staining nucleus of hepatocytes with *Ki67* antibodies (red arrow). (**F**) 5B2T group/6 weeks, shows negative staining nucleus of hepatocytes with *Ki67* antibodies (red arrow).

**Figure 6:**
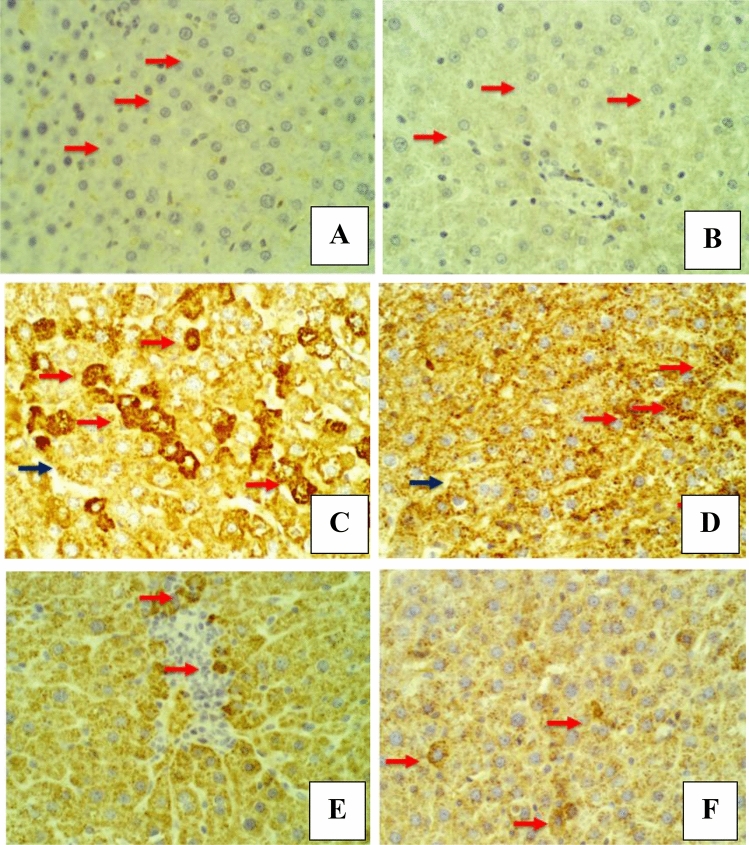
Liver section, IHC *HSA* -ab. 400×. (**A**) Placebo group/3 weeks, shows negative staining with *HSA* antibodies in the cytoplasm of hepatocytes (red arrow). (**B**) Placebo group/ 6 weeks, shows negative staining with *HSA* antibodies in the cytoplasm of hepatocytes (red arrow). (**C**) DEN/3 weeks, shows strong positive staining with *HSA* antibodies in the affected hepatocytes which stained as cytoplasmic golden brawn granule (red arrow), notice unspecific staining in other hepatocytes (black arrow). (**D**) DEN group/6 weeks, shows strong positive staining with *HSA* antibodies in the affected hepatocytes which stained as cytoplasmic golden brawn granule (red arrow). (**E**) 5B2T group/3 weeks, shows few weakly positive staining with *HSA* antibodies in the cytoplasm of hepatocytes as golden brown granules (red arrow). (**F**) 5B2T group/6 weeks, shows few weakly positive staining with *HSA* antibodies in the cytoplasm of hepatocytes as golden brown granules (red arrow).

The immunohistochemical changes were evaluated by screening the expression of two liver markers: Ki-67 and HSA. The findings of the placebo group (group I) showed negative staining with Ki-67 antibodies in the nucleus of the hepatocytes at 3 and 6 weeks (Fig. 5A and B). In contrast, the DEN-treated rats (group II) showed positive staining with Ki-67 antibodies in the affected hepatocytes for the first and second durations, and the affected cell nuclei were stained dark brown (Fig. 5C and D). The stained hepatic histological sections from 5B2T-treated rats (group III) showed weak positive staining with Ki-67 antibodies in the cytoplasm of the affected hepatocytes as golden-brown granules for the first duration (3 weeks); on the other hand, rat histological sections for the second duration (6 weeks) showed weak positive staining with Ki-67 antibodies in the cytoplasm of the injured liver cells and a golden-brown granule appearance (Fig. 5E and F). Moreover, the sections of the 10% Tween 80-treated rats (group I) showed negative staining with HSA antibodies in the cytoplasm of the hepatocytes for the first and second duration (Fig. 6A and B). The histological sections of DEN-treated rats (group II) showed strong positive staining with HSA antibodies in the affected hepatocytes, which stained as golden-brown cytoplasmic granules for the first and second durations, and the affected cell nuclei were stained dark brown (Fig. 6C and D). The stained hepatic histological sections of the 5B2T-treated rats (group III) showed weak positive staining with HSA antibodies in the cytoplasm of the affected hepatocytes as golden-brown granules for the first duration of the experiment (3 weeks); on the other hand, for the second duration (6 weeks), histological sections revealed weak positive staining with HSA antibodies in the cytoplasm of the injured liver cells with golden-brown granule appearance (Fig. 6E and F).

**Figure 7:**
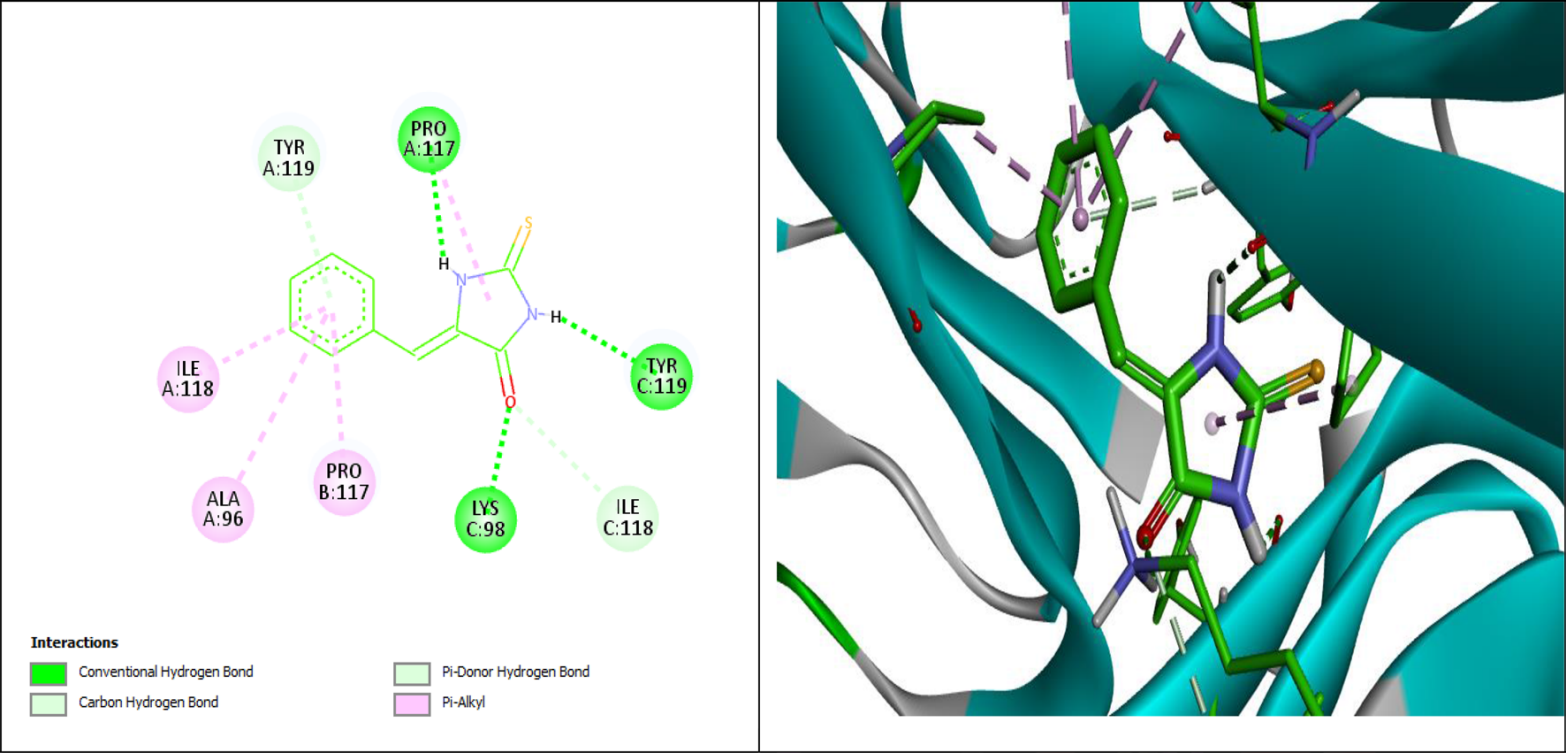
2D and 3D representation of the interaction of 5B2T with TNF-α (PDB ID: 1TNF).

**Figure 8:**
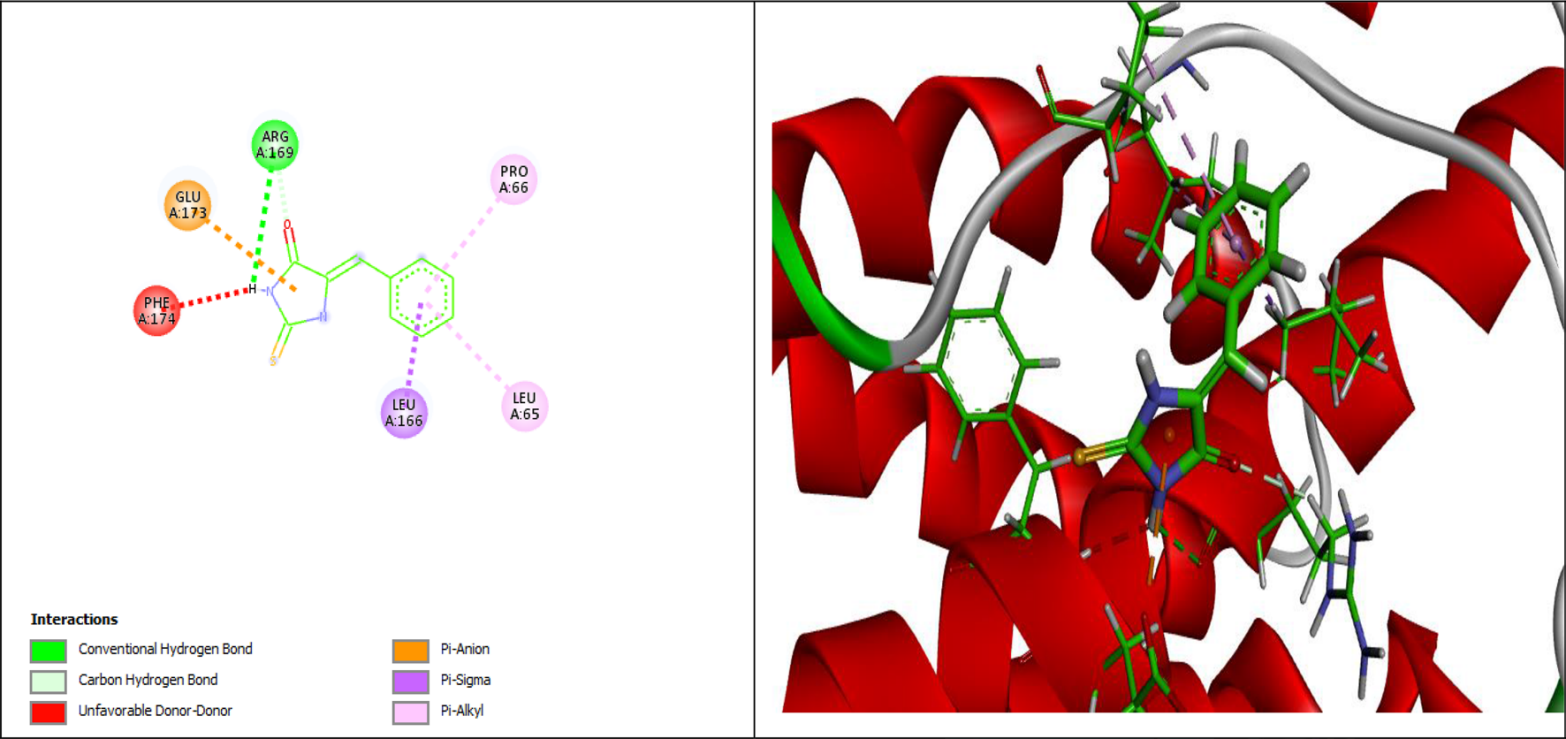
2D and 3D representation of the interaction of 5B2T with IL-6 (PDB ID: 1IL6).

The correct absorption and binding between the drug and the receptor is referred to as molecular docking. The most significant interaction of a ligand and receptor has the lowest docking energy. AutoDock Vina was used to evaluate the affinity, the conformation of binding, and the best ligand. For both of the ligands, among multiple docking poses, only the highest docking scores were included in the study. All the data regarding the binding force, number of hydrogen bond interactions and amino acid participation in the interactions that have been observed in TNF-α and IL-6 are listed in Table 3 and Figs. 7 and 8.

**Table 3:** Molecular docking of 5B2T.

Name	Binding Energy (kcal/mol)	Predicted Inhibition Constant p*K*i (µM)	Interaction position	Distance A°	Bonding type
TNF-α (PDB ID:1TNF)	− 6.7	5.9	PRO A: 117	2.36 (NHCS)	Conventional Hydrogen Bond
			TYR C:119	2.62 (OCNHCS)	
			LYS C:98	2.51CO	
			TYR A: 119	3.04	Pi-Doner H-bond
			PRO A:117	4.44	Pi-Alkyl
			ILE A:118	5.41	Pi-Alkyl
			ALA A:96	4.99	Pi-Alkyl
			PRO B: 117	3.91	Pi-Alkyl
			ILE C:118	3.32	Carbon-Hydrogen Bond
IL-6 (PDB ID: 1IL6)	− 6.1	5.4	ARG A:169	2.91	Conventional Hydrogen Bond
			ARG A:169	2.55	Carbon-Hydrogen Bond
			LEU A:166	2.47	Pi-Sigma
			LEU A:65	5.09	Pi-Alkyl
			PRO A:66	4.95	Pi-Alkyl
			GLU A: 173	3.88	Pi-Anion
			PHE A:174	2.42	Doner-Doner

5-benzylidine-2-thiohydantoin (5B2T) binds with TNF-α (PDB ID:1TNF) forming; hydrogen bonds with PRO A: 117, TYR C:119: 116, LYS C:98, TYR A: 119, ILE C:118 and with IL-6 (PDB ID: 1IL6) with ARG A:169, respectively. While, the hydrophobic interactions were observed between 5-benzylidine-2-thiohydantoin (5B2T) binds and TNF-α (PDB ID:1TNF) and IL-6 (PDB ID: 1IL6) with PRO A:117, ILE A:118, ALA A:96, PRO B: 117, LEU A:166, LEU A:65 and PRO A:66, respectively. Pi-anion interaction formed with GLU A: 173 and Unfavorable doner-doner bond was observed between 5B2T and PHE A; 174 amino acid in IL-6 (PDB ID: 1IL6) (Figs. 7 and 8).

In silico ADME/T and drug-likeness prediction of 5B2T was theoretically calculated via admetSAR and SwissADME. The molecular weight 204.25 (g/mol) has acceptable ADMET range properties. A significant value of 78% revealed a high chance of crossing blood brain barrier. The percentage of human intestinal drug absorption 98.73% was in the acceptable range (> 80). Octanol–water partition coefficient (Log P) of 1.03 was found to be less than 5 with no more than one violation is allowed. The topological surface areas (TPSA) were found to be in the acceptable range (< 140). In addition, H-bond acceptors (HBA) and donors (HBD) were found to be in the range of 3–6 and 2–4, respectively (Table 4).

**Table 4:** List of ADME and physicochemical properties of 5B2T.

MW (g/mol)	BBB+	Caco2+	HIA+	logp	TPSA A2	nON	nOHNH	RBs	N Violations	AMES toxicity	Carcinogenicity
180–500	− 3 to 1.2	< 25 poor > 500 great	< 25 poor > 80 high	< 5	≤ 140	2.0–20.0	0.0–6.0	≤ 10	< 5	Nontoxic	Non carcinogenic
204.25	0.78	93.81	98.73	1.03	73.22 Å	2	2	1	0	Nontoxic	Non carcinogenic

MW: molecular weight; BBB+ : blood–brain barrier; Caco2 + , Caco-2: Permeability; HIA + : %Human Intestinal Absorption; logp: logarithm of partition coefficient between n-octanol and water; TPSA2: topological polar surface area; nON: number of hydrogen bond acceptors; nOHNH: number of hydrogen bond donors; RBs: number of rotatable bond.

## Discussion

In the present study, we synthesized (Z)-5-benzylidene-2-thioxoimidazolidin-4-one under Knoevenagel condensation conditions. 1H-NMR spectrum for 5B2T showed a typical singlet signal for the hydrogen on the double bond (CH=C) at δ 6.49 ppm, and the three aromatic protons were displayed at δ 7.45–7.37 ppm as multiple signals and two aromatic protons were displayed as doublet at δ7.74 ppm (d, J = 7.4 Hz, 2H, phen). No overlaps were seen in 1H-NMR spectrum between a typical signal for the hydrogen on the double bond and aromatic ring signals for 5B2T. The 13C-NMR spectrum exhibited compatible signals with numbers of carbons presents in 5B2T. On the other hand, 13C-NMR spectrum indicated the presence of one stereoisomer which was represented by one carbon signal of each carbon atom in 5B2T. Under Knoevenagel condensations, E and Z geometrical isomers were possible during Knoevenagel condensations. While, 13C-NMR spectrum suggested one isomer of 5B2T and the configuration of Z-isomers suggesting that this configuration has greater thermodynamic stability due to less steric hindrance between the carbonyl group and CH = phenyl ring^21^.

DEN is a well-known carcinogenic chemical and is an acute hepatic toxin to many experimental animals. Prolonged administration of DEN has been proven to initiate hepatic tumors. A single intraperitoneal injection of 200 mg/kg DEN into experimental rodents can effectively induce irreversible liver injury^22^. The main factor that enables DEN to induce liver injury and cancer is the high possibility of this substance generating reactive oxygen species (ROS) that result in oxidative stress and damage to DNA, lipids and proteins. For DEN to be able to establish these actions, it must be metabolized first by an enzyme inside the body called cytochrome p450, which makes DEN produce high levels of ROS that lead to lipid peroxidation of the cell membrane and DNA adducts via alkylation, resulting in cell damage and injury. The successful administration of DEN in experimental animals results in 100% induction of hepatocellular carcinoma (HCC)^23^. In this study, a single intraperitoneal injection of 200 mg/kg DEN was employed to induce liver injury and cancer in experimental animals/male rats to evaluate the hepatoprotective effects of 5B2T (a drug-like molecule) in treating hepatic disorders. The results of the biochemical analysis are shown in Fig. 2, in which it is obvious that administration of DEN caused a noted serum elevation in the levels of liver biochemical markers, including aminotransferases (ALT, AST and ALP), as an indication of liver injury and instability in hepatic metabolic activities. This elevation may be attributed to the cytoplasmic release of these enzymes into the blood circulation following the rupture of the plasma membrane due to the cellular damage induced by DEN^24^. Alkaline aminotransferase (ALT), aspartate aminotransferase (AST) and alkaline phosphatase (ALP) are known to be the most sensitive serum biochemical biomarkers for the diagnosis of any hepatic disability^25^. The oral supplementation of 5B2T showed a potent decline in the levels of the serum enzymes that were previously induced by DEN. Many studies supported these results and reported the same high levels of biochemical markers of the liver during DEN-induced carcinogenesis, including a study evaluating garlic oil against HCC induced by DEN^26^. This suggests the ability of 5B2T to inhibit tumor progression in rats induced by DEN, which may be attributed to the capability of the used treatment to preserve the unity and integrity of the cell's plasma membrane, preventing the leakage of these cytoplasmic enzymes from the inside of the cells to the outside through membranes and expressing hepatoprotective activities. This may also be the reason why the biochemical markers restored their activity after the administration of 5B2T also other factors may contributed including the metal type, the ligand type and its donor atoms. Alongside the biochemical evaluation, this study also included the evaluation of the enzyme-linked immune sorbent essay (ELISA) technique for further illustrations of the effects of both the inducer and the treatment on the liver. Figure 3 shows the effect of 5B2T on proinflammatory TNFα and IL-6 cytokine levels. A strong increase was observed in the serum levels of proinflammatory cytokines in rats treated with DEN, and these results were associated with cancer and inflammation (especially TNF-α and IL-6), which have been documented to be increased after the administration of DEN^27^. In contrast, rats that were treated with 5B2T revealed a notable decrease in the levels of these cytokines. TNF-α mediate cytotoxic effects by expression of p55 and p75 receptors in his molecular mass of 55 kDa, Thus inducing of (ROS) reactive oxygen species in their cell membrane’s nicotinamide adenine dinucleotide phosphate and endothelial mitochondria also TNF-α disrupt the electron transport chain in the mitochondrion complex beside stimulation of nuclear factor-kappa beta activation and up regulation of IL-6 expression^28^.

**Figure 9:**
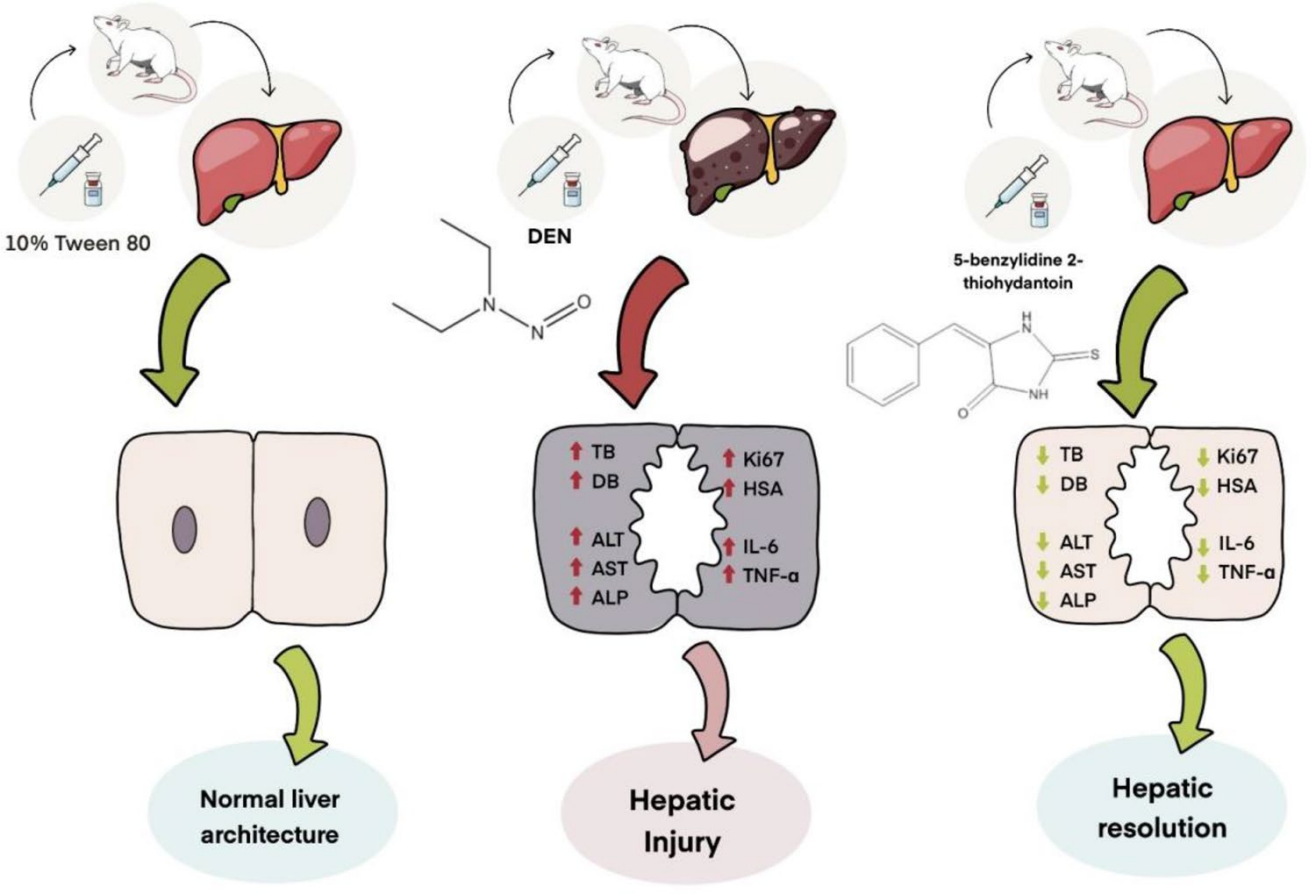
The effect of 5-benzylidine-2-thiohydantoin on liver injury induced by diethylnitrosamine. DEN: Diethylnitrosamine, TNFα: tumor necrosis factor alpha and IL-6: Interleukin 6. TB: total bilirubin, DB: direct bilirubin, AST: acetate aminotransferase, ALT: alanine aminotransferase, ALP: alkaline phosphatase.

These findings were supported by the histopathological and immunohistochemical improvements in the tested liver sections. Following the administration of DEN, the characteristic histological features of the liver were distorted, disorganized and lost. This may occur because of a series of reactions, including oxidative stress, loss of cell membrane integrity, infiltration of inflammatory cells and the eventual transformation of normal hepatocytes into tumor cells^29^. Figure 4 demonstrates the histopathological results. Microscopic results of the histopathologically tested liver sections taken from DEN-treated rats in the first duration of the experiment revealed massive disfiguration and damage to the liver, mainly represented by portal area fibrosis and dilatation in the sinusoids. On the other hand, the 6-week duration of DEN administration showed a more serious and advanced stage of liver injury indicated by infiltration of inflammatory cells in the central vein area, bile canaliculi dilatation, peliosis and adenoma, supporting the long-term destructive effects of DEN on the liver. The same results were found in a study evaluating the effect of ginger against DEN-induced hepatotoxicity in rats^30^, supporting the harmful effects of DEN on the liver. In contrast, liver sections of the 3-week-treated rats with 5B2T showed improvements in the liver's general architecture, with fewer fibrotic and inflammatory cells. At the same time, rats that were treated for 6 weeks with 5B2T not only showed inhibition of the destructive effects of DEN but also showed restoration of partial to complete histological features to a greater extent than the first duration of treatment. Additionally, immunohistochemical evaluation of two main liver-specific antibodies, Ki-67 and HSA, is illustrated in Figs. 5 and 6. The placebo group showed negative staining of Ki-67 and HSA in the nucleus of the tested tissue cells. The tissue sections of DEN-treated rats revealed strong positive staining for Ki-67 and HSA antibodies for both experiment durations, which aggressively supports the pernicious effects of DEN on hepatocytes. In a study carried out to evaluate Ki-67 in DEN-induced HCC in rats, a significant increase in the number of Ki-67-positive cells was observed after DEN induction^31^. Treatment with 5B2T showed interesting results that improved the prognosis, reflecting the high efficacy of the chemical in curing an advanced stage of liver injury. The tested liver sections for HSA from the 3- and 6-week-treated rats demonstrated weak positive staining in the cytoplasm of the affected hepatocytes. Comparing these results to the Ki-67 evaluation, more interesting results were found suggesting that a longer treatment duration has a direct proportional relationship with the increased efficacy of the drug, regardless of the administered dosage (i.e., with the stability of the drug dose). The tested sections of the 3-week-treated rats with 5B2T showed few positively stained nuclei with Ki-67 Abs; in contrast, the 6-week treatment duration results showed total negative staining with Ki-67 Abs. The noted curing abilities of this unique derivative of the thiohydantoin group of drug-like molecules may be attributed to its possession of a stereogenic center in its 5th position^32^. Nevertheless, it is also believed that these chemical derivatives of thiohydantoin are able to block the liver's inflammation and injury receptors, as well as inhibit their expression. For example, it is believed that 2-thiohydantoin derivatives have a major role in the inhibitory activity of important enzymes, such as nicotinamide adenine dinucleotide phosphate (NAPDH) oxidase (NOX), which has an important role in the Krebs cycle and in ranging host defense to inflammation as well as cell signaling, and isocitrate dehydrogenase (IDH), which has a pivotal role in the tricarboxylic acid cycle^10^. The thiohydantoin group is widely known for its pharmacological uses as antimicrobial and anticancer agents, especially 2-thiohydantoin derivatives, because of their low toxicity to human cells. As mentioned before, the 5B2T derivative is a valuable and promising option that can be used in the future as a treatment for liver disorders due to its easy preparation and, more importantly, the possession of a stereogenic center at the fifth position^33^. The highest binding site (− 7.1 kcal/mol) suggests a strong binding site affinity for the 5B2T interaction with TNF-α. 5B2T establishes three hydrogen bonds with GLU B161, GLU C: 116, and PRO100 and one hydrophobic bond with GLU173. The IL-6 (PDB ID: 1IL6) binding site and 5B2T interaction has a significant binding site energy of -6.1 (Kcal/mol) and establishes one hydrogen bond with SER170 and three hydrophobic bonds with LEU 65, 166, and PRO 66. The ADMET analysis for 5B2T was found to be compatible with the rule of five (RO5) for drug-like molecule according to Lipinski and his team^34^. The molecular weight is < 500/gmol which is obey with the reference range. Blood–brain barrier (BBB) + value describes the ability of the compound to cross the BBB, which is in the permissible ranges. The topological surface areas (TPSA), log *p*, H-bond acceptors (HBA) and donors (HBD) were found to be in acceptable range. The values show that 5B2T can be absorbed by human intestine, and it is nontoxic and non-carcinogenic compound. All values revealed that 5B2T satisfies with the rules of Lipinski’s rule of five (Ro5), and Veber, 4. The promising results for 5B2T indicate that they can be used as drug candidates. In this study, single intraperitoneal injection of 200 mg/kg of DEN was employed to induce liver injury and cancer in experimental animals/male wistar rats, for the evaluation of the hepatoprotective effects of 5-benzylidine-2-thiohydantoin chemical (drug-like molecule) in treating hepatic disorders, Fig. 9 summarizes the experiment. Administration of 10% of Tween 80% was considered to be the study's control. Rats treated with DEN caused a massive liver injury that’s in particular caused elevation of liver's biochemical markers (total bilirubin, direct bilirubin, indirect bilirubin, alanine aminotransferase, aspartate aminotransferase and alkaline phosphatase) along with pro-inflammatory cytokines elevation (tumor necrosis factor-alpha and interleukine-6) and a detected high levels of HAS and ki-67 antibodies, eventually this resulted in loss of hepatic architecture. Rats treated with 5-benzylidine-2-thiohydantoin notably restored hepatic functions and architecture that was represented in the decrease of the same hepatic markers. These results strongly support the gold therapeutic characteristics of the treatment, and promising hepatoprotective activities.

## Conclusion

Experimentally, the synthesized chemical (5-benzylrdine-2-thiohydantoin) clearly established curative properties against induced liver injury represented by liver biochemical markers, pro-inflammatory cytokines (TNF-α and IL-6), immuno histochemistry findings. Also molecular docking results showed strong binding with high energy at different sites between the 5-benzylidine-2-thiohydantoin and pro-inflammatory cytokines, showing the efficacy of the chemical as liver treatment.

## Data Availability

The datasets used and/or analyzed during the current study are available from the corresponding author on reasonable request.
